# Supplementary figures and images for: Comprehensive Transcriptome Analysis in the Testis of the Silkworm, Bombyx mori
Source: Insects. 2023 Aug 2;14(8):684. doi: 10.3390/insects14080684 (PMC10455414; doi:10.3390/insects14080684)

## Slide 1
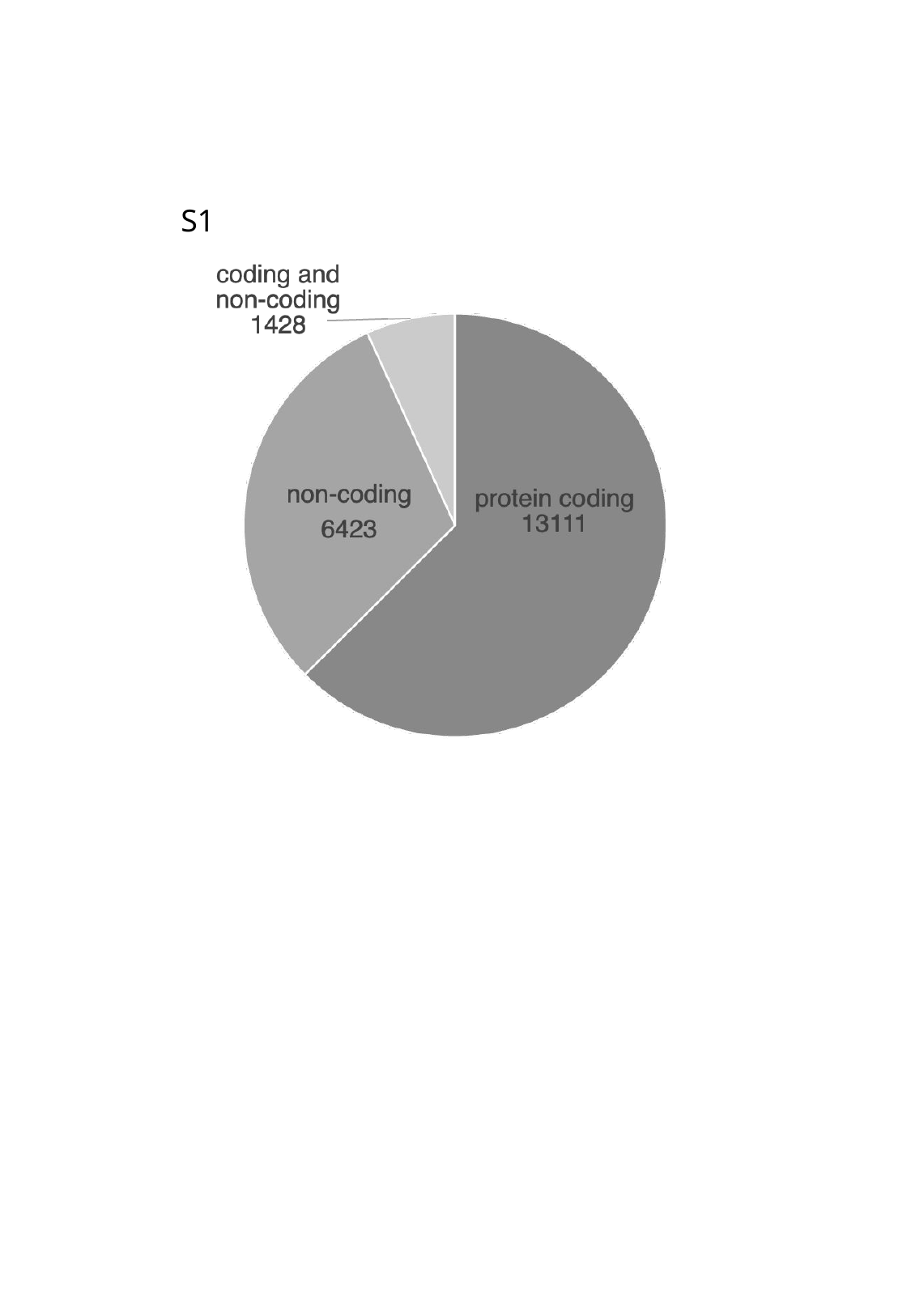

S1

## Slide 2
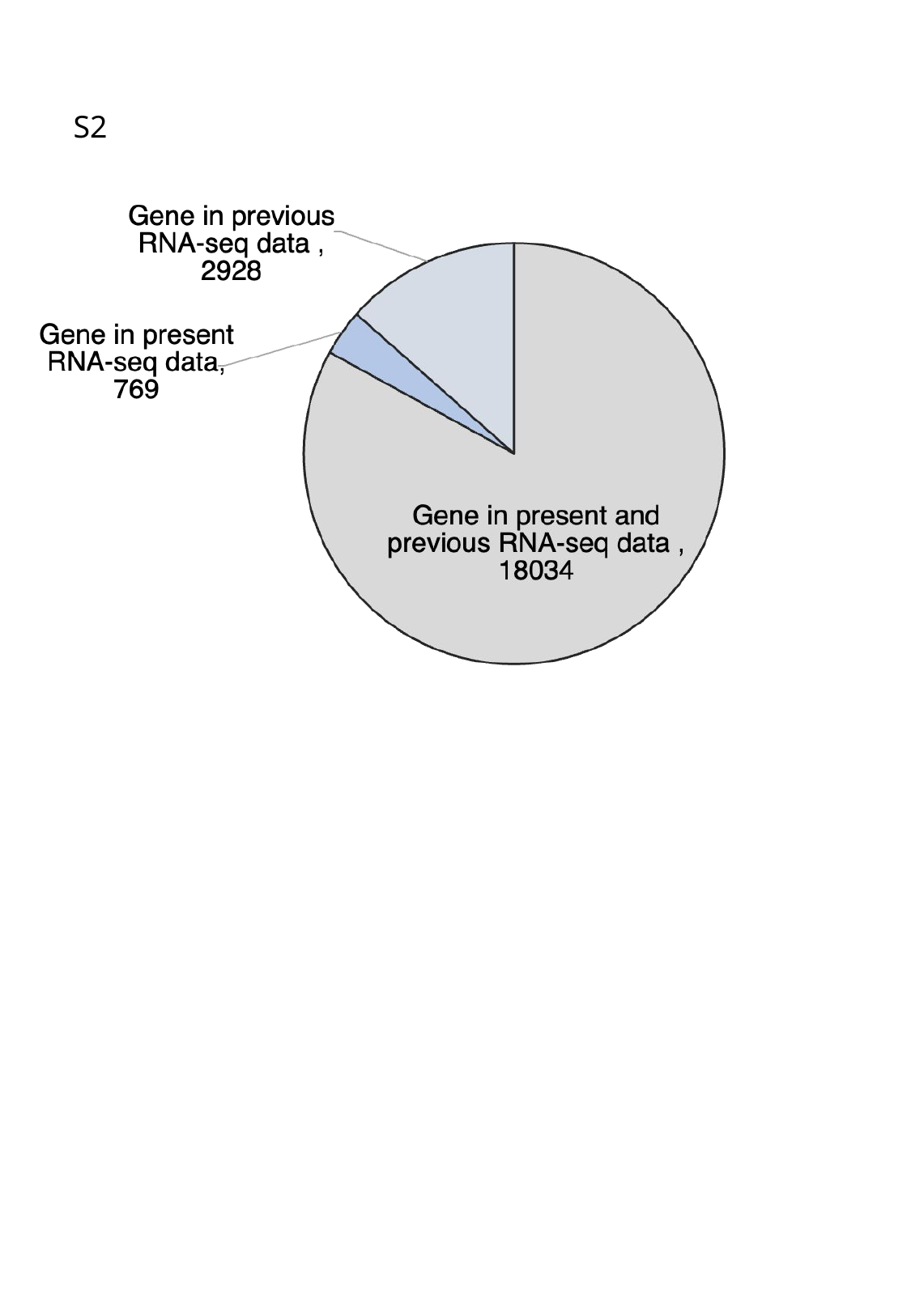

S2

Supplement: Supplementary file 1 [file insects-14-00684-s001.zip › supplementary data/supplementary figure.pptx]
